# Genome Wide Transcriptome Analysis Reveals Complex Regulatory Mechanisms Underlying Phosphate Homeostasis in Soybean Nodules

**DOI:** 10.3390/ijms19102924

**Published:** 2018-09-26

**Authors:** Yingbin Xue, Qingli Zhuang, Shengnan Zhu, Bixian Xiao, Cuiyue Liang, Hong Liao, Jiang Tian

**Affiliations:** 1 Root Biology Center, State Key Laboratory for Conservation and Utilization of Subtropical Agro-Bioresources, College of Natural Resources and Environment, South China Agricultural University, Guangzhou 510642, China; yingbinxue@yeah.net (Y.X.); 15889964001@163.com (Q.Z.); shnzhu@163.com (S.Z.); 13570450400@163.com (B.X.); liangcy@scau.edu.cn (C.L.); 2 Root Biology Center, Haixia Institute of Science and Technology, Fujian Agriculture and Forestry University, Fuzhou 350000, China; hliao@fafu.edu.cn

**Keywords:** RNA-seq, nodule, P deficiency, symbiotic nitrogen fixation, soybean

## Abstract

Phosphorus (P) deficiency is a major limitation for legume crop production. Although overall adaptations of plant roots to P deficiency have been extensively studied, only fragmentary information is available in regard to root nodule responses to P deficiency. In this study, genome wide transcriptome analysis was conducted using RNA-seq analysis in soybean nodules grown under P-sufficient (500 μM KH_2_PO_4_) and P-deficient (25 μM KH_2_PO_4_) conditions to investigate molecular mechanisms underlying soybean (*Glycine max*) nodule adaptation to phosphate (Pi) starvation. Phosphorus deficiency significantly decreased soybean nodule growth and nitrogenase activity. Nodule Pi concentrations declined by 49% in response to P deficiency, but this was well below the 87% and 88% decreases observed in shoots and roots, respectively. Nodule transcript profiling revealed that a total of 2055 genes exhibited differential expression patterns between Pi sufficient and deficient conditions. A set of (differentially expressed genes) DEGs appeared to be involved in maintaining Pi homeostasis in soybean nodules, including eight *Pi transporters* (*PTs*), eight genes coding proteins containing the *SYG1/PHO81/XPR1* domain (*SPXs)*, and 16 *purple acid phosphatases* (*PAPs*). The results suggest that a complex transcriptional regulatory network participates in soybean nodule adaption to Pi starvation, most notable a Pi signaling pathway, are involved in maintaining Pi homeostasis in nodules.

## 1. Introduction

Phosphorus (P) is an essential plant macronutrient. As a key component of biomolecules, such as nucleic acids, proteins, and phospholipids, P is involved in multiple biosynthetic and metabolic processes throughout plant growth and development [1,2]. Phosphate (Pi), the major form of phosphorus acquired by plants, is not only unevenly distributed in soils, but is also readily fixed onto soil particles into unavailable forms (e.g., aluminum-P, iron-P, and calcium-P) [3,4,5]. Low P availability significantly decreases crop yields, and, thus, becomes a major constraint on crop growth and production [6]. At the other end of the spectrum, excessive application of P fertilizer is inadvisable, due to depletion of limited P rock resources and eutrophication of marine systems by Pi in runoff that is not utilized by plants [6,7]. Intelligent use of moderate amounts of P fertilizer can be beneficial if crops are developed for such conditions. To meet these goals of developing smart crop cultivars with high P utilization efficiency requires further understanding of genetic and molecular mechanisms underlying plant adaptions to P deficiency [8,9,10,11,12].

To date, a range of morphological, physiological and molecular processes have been associated with plant in adaptation to P deficiency. These processes include the remodeling of root morphology and architecture, increased exudation of organic acids and acid phosphatases, enhanced expression of Pi transporters, and formation of symbiotic interactions with mycorrhizal fungi or rhizobia [13,14,15,16,17,18,19]. In recent years, many Pi starvation responsive genes and proteins have been identified and functionally characterized, which has filled in large gaps in our sketch of plant Pi signaling and regulatory networks [5,9,11]. In the center of the Pi signaling network lie several important regulators, such as *phosphate starvation response 1* (*PHR1*) and WRKY transcription factors, proteins containing the SYG1/PHO81/XPR1 domain (SPX), and the ubiquitin-like modifier E3 ligase [20,21,22,23,24,25]. Downstream responses include a set of genes directly involved in morphological and physiological responses to Pi starvation, such as *purple acid phosphatase* (*PAP*) genes functioning in extracellular organic P remobilization, *phosphate transporter* (*Pht*) genes involved in Pi acquisition, and *expansin* (*EXP*) genes that participate in alteration of root morphology and architecture [26,27,28,29,30].

Among all of the genes, *Pht* genes are widely characterized in plants, especially in rice (*Oryza sativa*) and *Arabidopsis thaliana*. It has been documented that transcripts of a set of *Pht* genes were increased by Pi starvation, such as 4 of 9 *Pht genes* in Arabidopsis and 4 of 13 in rice [31,32]. Furthermore, *AtPht1;1* and *AtPht1;4* are suggested to be responsible for about 50% of Pi uptake under Pi starvation conditions in Arabidopsis [33,34]. Similarly, *OsPht1;1*, *OsPHT1;9* and *OsPHT1;10* were found to modulates phosphate uptake and translocation [35,36,37]. Recently, several *Pht* genes have been suggested to regulate root growth, such as *AtPht1;5* for root hair formation and primary root growth in Arabidopsis, *OsPht1;8* for adventitious root elongation and lateral roots number [38,39]. Another critical gene family in regulating Pi homeostasis, *SPX* family, has also been well characterized in plants. In Arabidopsis, transcripts of 3 *AtSPX* members were enhanced by P deficiency except to *AtSPX4* [22]. Similar to Arabidopsis, 5 *OsSPX* members were up-regulated by P deficiency except to *OsSPX4* [40]. Recently, a highly conserved mechanism has been suggested that SPX proteins might act as an intracellular Pi sensor mainly through interactions with PHR1/PHR2 in both rice and Arabidopsis [41,42,43,44,45].

Additionally, plant phytohormones are suggested to regulate plant responses to Pi starvation, such as auxin, abscisic acid, ethylene, cytokinin [9,46,47]. Furthermore, cross-regulation also occurs between Pi and nitrogen (N) starvation in both legume and non-legume plants [48,49]. For example, a critical regulator for Arabidopsis adaptation to nitrogen availability, N limitation adaptation (NLA) was suggested to regulate Pi homeostasis by recruiting PHOSPHAT2 (PHO2) to degrade Pht1;4 in Arabidopsis [48]. Furthermore, transcription levels of *NLA* were found to be regulated by a Pi starvation responsive *miR827* [49], strongly suggesting there is a crosstalk between N and P deficiency. For legume plants, a crosstalk between N and P deficiency could be directly reflected by significant decreases of both N_2_ fixation capability and growth in legume nodules by Pi starvation [28,50,51]. However, one outstanding issue is that a large fraction of our knowledge of Pi signaling networks has been attained in model plants, such as *Arabidopsis thaliana* and rice (*Oryza sativa*). Verification and application of this knowledge remain fragmentary for most crops, particularly legume crops.

Soybean (*Glycine max* L.) is an important legume crop that is a source of high-quality protein and oil [52]. Similar to other legumes, soybean participates in symbiosis with rhizobia in the formation of nodules [53]. It has been well documented that rhizobium establishment is a complex process, which is mainly regulated by phytohormones, such as auxin, cytokinin, ethylene, gibberellic acid, strigolactones, jasmonic acid, abscisic acid, and salicylic acid [54,55]. For example, it has been suggested that ethylene negatively regulates rhizobia infection and nodule organogenesis because suppression of both *LjEIN2a* and *LjEIN2b* led to a hypernodulation phenotype in *Lotus japonicus* [56]. Recently, gibberellic acids have been suggested to negatively regulate root nodule symbiosis in *Lotus japonicus* and *Medicago truncatula* [57,58,59]. Furthermore, it has been documented that P availability adversely affects soybean nodule development and growth [60,61,62]. In addition, it has been suggested that responses to P deficiency are similar between roots and nodules, because of significant increases of proton exudation, and, thus, decreases of rhizosphere pH were observed in soybean grown in low P conditions [18,63]. However, few studies were conducted to investigate gene expression patterns between nodules and roots in legume crops. For example, it has been documented that 11 of 14 *GmPT* members exhibited Pi-starvation responsive expression patterns in soybean roots [28,29], but information about transcripts of all *GmPT* members responsive to Pi-starvation remains largely unknown in soybean nodules. Recently, increased transcription of *GmPT5* has been shown to play a critical role in maintaining Pi homeostasis in soybean nodules [62]. Plus, a Pi starvation responsive gene, *GmEXPB2*, plays vital roles in adaptive responses of both soybean roots and nodules to P deficiency, possibly through cell wall modifications [26,51]. The results strongly suggest that identification and functional analysis of Pi starvation responsive gene is critical for elucidating adaptive strategies to low P stress in soybean nodules. Yet, genome-wide transcriptome analysis has not been conducted to identify Pi starvation responsive genes in soybean nodules.

Although genome-wide transcriptome analysis has been successfully used to elucidate molecular mechanisms underlying complex adaptations of plants to P deficiency using RNA-seq technique, most of these studies focus on roots or leaves of plants grown under non-symbiotic conditions. Little transcriptome information is available for legume nodules. As far as the authors are aware, only three studies have been conducted to investigate global gene expression responses to Pi starvation in legume nodules, including with bean (*Phaseolus vulgaris*), *Medicago truncatula* and chickpea (*Cicer arietinum*) [64,65,66]. However, there is little information about genome-wide analysis of gene transcripts responsive to Pi starvation in soybean nodule. Furthermore, it is well known that the formation of symbiotic nodules and their responses to Pi starvation varies considerably among legume species and rhizobium strains [66,67]. Thus, it is important to investigate molecular mechanisms underlying nodule development and physiology for each commercially important legume crop under Pi starvation.

In this study, genome-wide transcriptomic analysis of soybean nodules in response to P deficiency was conducted via RNA-seq. Thousands of differentially expressed genes were identified in soybean nodules under P deficiency, with many involved in nutrient/ion transport, transcriptional regulation, key metabolic pathways, Pi remobilization, and signaling. These results will enable future researchers to further elucidate molecular processes within nodules adapted to P deficiency, which will ultimately lead to the development of P-efficient soybean varieties that can maintain symbiotic nitrogen fixation (SNF) in low or moderate P availability systems.

## 2. Results

### 2.1. Growth of Soybean Nodules Is Inhibited by Pi Starvation

**Figure 1:**
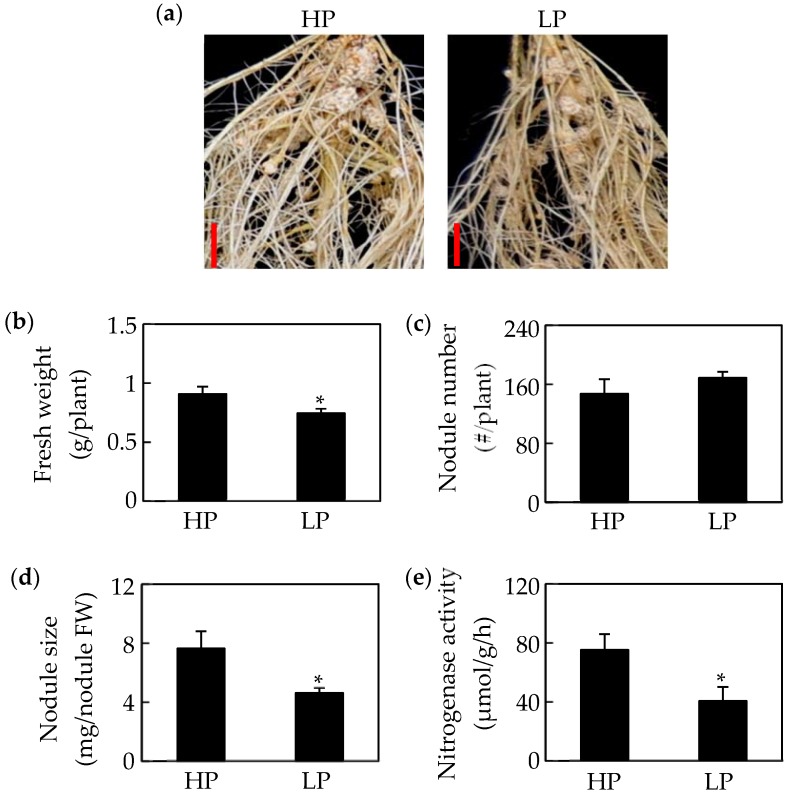
Effects of phosphorus (P) deficiency on soybean nodule growth. (**a**) Phenotype of soybean nodules at two P levels. (**b**) Soybean fresh weight. (**c**) Nodule number. (**d**) Nodule size. (**e**) Nodule nitrogenase activity. Data in the figure are mean of four replicates with standard error bars. Asterisks indicate significant difference between HP (500 μM KH_2_PO_4_) and LP (25 μM KH_2_PO_4_) treatments in the Student’s *t*-test (*: *p* < 0.05). Bars = 1 cm.

**Figure 2:**
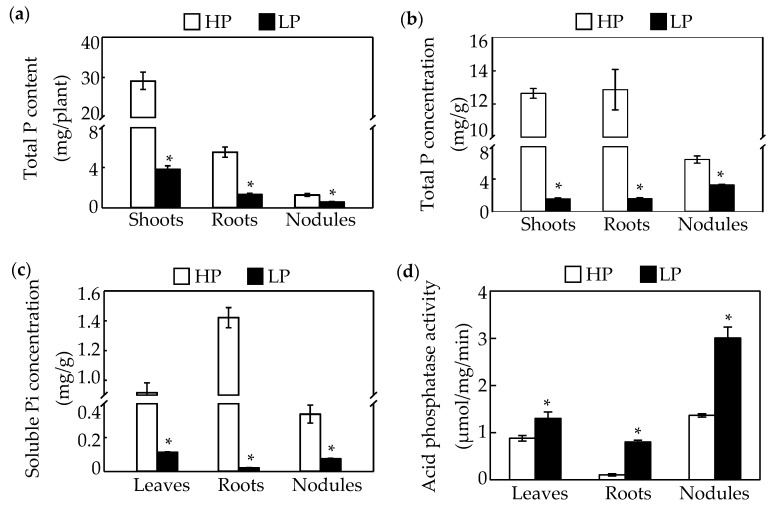
Effects of P deficiency on phosphate (Pi) accumulation and acid phosphatase (APase) activity in soybean. (**a**) Total P content of shoots, roots, and nodules. (**b**) Total Pi concentrations in shoots, roots, and nodules. (**c**) Soluble Pi concentrations in leaves, roots, and nodules. (**d**) Acid phosphatase activity of leaves, roots, and nodules. Data in the figure are mean of four replicates with standard error bars. Asterisks indicate significant difference between HP and LP treatments in the Student’s *t*-test (*: *p* < 0.05).

Phosphate starvation significantly affects soybean nodule growth. The results in this study showed that nodule size, fresh weight, and nitrogenase activity in nodules declined in response to Pi starvation by 27%, 36%, and 45%, respectively (Figure 1a,b,d,e). However, there was no significant difference between phosphorus treatments in nodule number (Figure 1c). Consistently, Pi starvation significantly reduced both P content and total P concentration of seedlings and nodules (Figure 2a,b). Compared to values in HP plants, the P content and total P concentration of shoots and roots decreased by more than 70%, whereas, the P content and total P concentration in nodules decreased only by 55% and 49%, respectively, under LP conditions (Figure 2a,b). Accompanying decreases in P content, soluble Pi concentrations also decreased significantly in response to Pi starvation, with observed soluble Pi concentrations declining by 88% in leaves, 98% in roots, and 77% in nodules (Figure 2c). In contrasting, acid phosphatase (APase) activity increased significantly with P deprivation, as reflected by 0.4, 6.9, and 1.2-fold increases in leaves, roots, and nodules, respectively (Figure 2d). Furthermore, the highest APase activities were observed in nodules, especially with low P availability, where this activity was 2.3 and 3.7 fold higher than in leaves and roots, respectively (Figure 2d).

### 2.2. Amino-N Compounds in Nodules Produced in Low P and High P Conditions

The amino acid/amide composition in HP or LP treated nodules was analyzed to check for effects of P availability on N metabolism. A total of 25 amino-N compounds were detected, and P deficiency significantly affected the concentrations of many, as well as, their relative proportions in soybean nodules (Table 1). Under P deficient conditions, the concentration of total amino-N compounds increased by 35% compared to that under P sufficient conditions. Closer inspection of individual compounds revealed that the P deficiency led to increased concentrations of six amino-N compounds, namely asparagine, glutamine, arginine, histidine, phosphoethanolamine and isoleucine, with the largest increase of 8.2-fold observed for phosphoethanolamine (Table 1). On the other side, the concentration of four amino-N compounds decreased significantly with Pi starvation, including glutamic acid, aspartic acid, β-Alanine, and cysteine (Table 1). Despite these differences, asparagine remained the most prevalent amino-N compound in soybean nodules, as reflected by this compound comprising 69% and 79% of the total amino-N compounds in HP and LP treated nodules, respectively (Table 1).

### 2.3. Changes of Transcriptomes in Soybean Nodules Resulting from Pi Starvation

Transcriptome analysis of soybean nodules in response to Pi starvation was determined by RNA-seq analysis. A total of six libraries were constructed. RNA-seq analysis produced about 47.6 and 48.1 million raw reads for nodules at HP and LP levels, respectively (Table S1). After excluding the low-quality readings, about 45.6 and 46.2 million clean reads were obtained for libraries of HP and LP nodules, respectively (Table S1). Among them, a total of 42.8 and 43.3 million clean reads perfectly mapped to soybean reference genes for each P treatments (Table S1). Finally, a total of 38,831 and 38,874 gene transcripts were found to be expressed in P-sufficient and P-deficient nodules, respectively (Table 2). Differentially expressed genes (DEGs) were those with transcripts observed to have at least 2-fold changes in expression, as well as, *q* value ≤ 0.05. Using these criteria, a total of 2055 genes were considered to be differently expressed in soybean nodules between the two P treatments (Table 2). Among them, 1431 genes were up-regulated by P deficiency, while 624 genes were down-regulated (Table 2 and Table S2). Gene ontology (GO) category analysis showed that the 2055 Pi-responsive genes could be divided among 22 biological processes, 14 cellular component, and 11 molecular function terms (Figure S1). Among these categories, cellular process, cell part, and binding function were the most prevalent among biological process, cellular component and molecular function terms, respectively (Figure S1).

### 2.4. Analysis of Pi-Responsive Genes Involved in Metabolome

**Figure 3:**
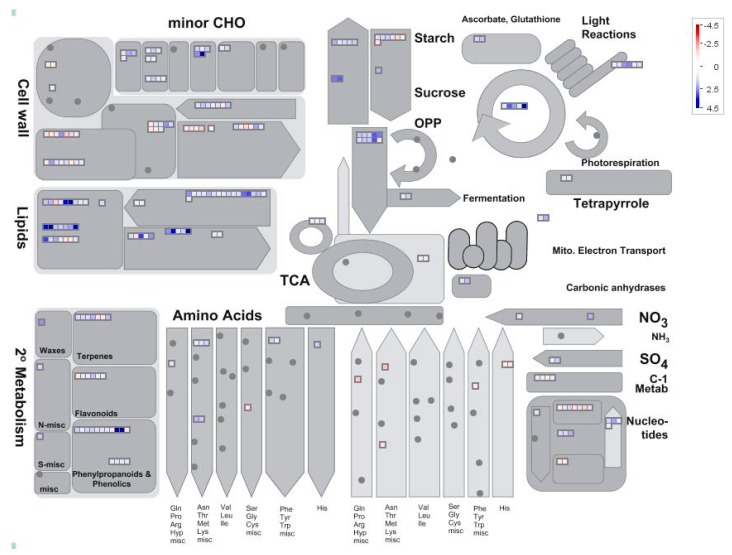
MapMan analysis of differentially expressed genes in soybean nodules. In the color scale, blue and red represent up-regulated and down-regulated expression, respectively, in response to Pi starvation within soybean nodules. Black dots represent no gene enrichment to the category entries. Numbers represent fold changes in expression levels expressed as Log_2_(LP/HP).

MapMan analysis was further used to examine DEGs in soybean nodules. The expression ratios of LP/HP were utilized and graphical representations were obtained for visual analysis from MapMan (Figure 3). In total, the differentially expressed genes were predicted to participate in 22 metabolic processes (Figure 3). However, Pi-starvation responsive genes were mainly associated with lipid metabolism (55 genes), hormone metabolism (46 genes), cell wall (44 genes), and secondary metabolism (31 genes). Of the remaining categories, 18, 17, 15, 13, 11, and 10 genes were respectively associated with minor CHO (carbohydrate) metabolism, nucleotide metabolism, major CHO metabolism, redox, photosynthesis process, and glycolysis (Figure S2). At the low end of representation, less than five genes were predicated to be involved in C1 (one carbon)-metabolism, TCA (Tricarboxylic Acid) transformation, gluconeogenesis/glyoxylate cycle, polyamine metabolism, mitochondrial electron transport/ATP synthesis, fermentation, tetrapyrrole synthesis, S-assimilation, N-metabolism, co-factor, and vitamin metabolism, and biodegradation of xenobiotics (Figure S2). Furthermore, 16 genes were identified to be involved in amino acid metabolism (Table S3). Among them, a total of nine genes were up-regulated by Pi starvation, including three *asparagine synthetases*, two *ornithine decarboxylases*, two *lysine decarboxylases*, one *glutamine synthetase*, and *glutamyl-tRNA (Gln) amidotransferase subunit C* (Table S3). However, a total of seven genes were down-regulated by Pi starvation, including two *histidine decarboxylases*, two *S-adenosylmethionine decarboxylases*, one *cysteine synthase*, *tyrosine aminotransferase*, and *lysine decarboxylase* (Table S3).

### 2.5. Identification of Pi-Starvation Responsive Genes Controlling Nodule Pi Homeostasis

Since soybean nodules are a P sink, three gene families involved in Pi homeostasis were further analyzed among DEGs, including *PT* (*Phosphate Transporter*), *PAP* (*Purple Acid Phosphatase*) and *SPX* (proteins containing the *SYG1/PHO81/XPR1* domain). Out of 14 *GmPT* members, eight were significantly enhanced in nodules in response to P deficiency (Table 3). Among them, more than 16-fold increases were observed for transcripts of *GmPT5* and *GmPT6* in P-deficient nodules (Table 3). Phylogenetic analysis showed that GmPT proteins encoded by eight Pi-starvation responsive *GmPT* members were classified into two sub-groups (Figure S4). GmPT1/4/7/13 were classified into sub-group I, with MtPT1/2/3/5 and AtPht1;1/2/3. However, GmPT2/5/6/14 belonged to sub-group II with AtPht1;4/7 (Figure S4). For *SPX* family, eight *GmSPX* members were significantly up-regulated in nodules by Pi starvation, especially *GmSPX9* with a 168-fold increase (Table 3). Phylogenetic analysis showed all proteins encoded by Pi-starvation responsive *GmSPXs* were classified into three sub-groups, including sub-group I, II, and V (Figure S5). GmSPX3/7/8 were classified into sub-group I, containing PvSPX1/2, and AtSPX1/2 (Figure S4). Sub-group II contained GmSPX2/4/5/9 and PvSPX3 (Figure S5). GmSPX1/10 belonged to sub-group V with AtSPX3 and OsSPX3/5/6 (Figure S5). For the *PAP* family, 16 members were significantly up-regulated, most of all, *GmPAP11* with a 41-fold increase in expression (Table 3). Phylogenetic analysis showed that all identified GmPAPs were classified into four sub-groups (Figure S6). GmPAP11/20/23 were classified into sub-group I, with PvPAP2, SgPAP10, AtPAP10, AtPAP12, OsPAP10a, and OsPAP10c (Figure S6). GmPAP21/31/32 belonged to sub-group III together with OsPAP21b/23, AtPAP15/23 (Figure S6). GmPAP1/17/30/35, together with OsPAP9b, AtPAP2/9 belonged to sub-group IV (Figure S6). GmPAP8/9/10/13/15/16 belonged to sub-group V with PvPAP3, SgPAP7 and AtPAP17 (Figure S6).

### 2.6. Identification of Genes Functioning as Transporters

**Figure 4:**
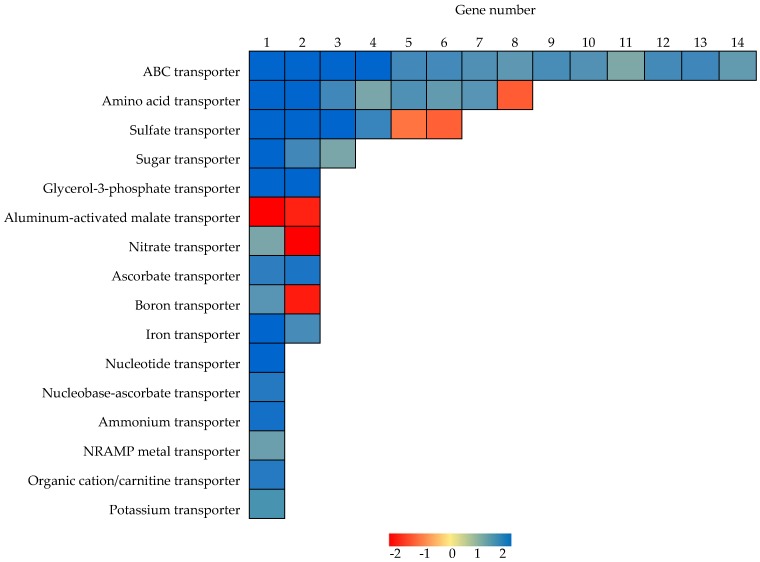
Heatmap analysis of P responsive DEGs associated with transport in nodules. Blue and red represent up-regulated and down-regulated expression, respectively, in response to Pi starvation within soybean nodules. Numbers represent fold changes in expression levels expressed as Log_2_(LP/HP).

In addition to Pi high affinity transporters, a total of 16 other types of transporters were also identified as responsive to P deficiency in soybean nodules (Figure 4 and Table S4). Among them, genes encoding ABC transporters were the most abundant, and then the amino acid transporters, with 14 and eight of each type, respectively, identified as P responsive in soybean nodules (Figure 4). Of these, all of 14 ABC transporters were up-regulated, while seven amino acid transporters were up-regulated and one was down-regulated (Figure 4).

In regard to transport of other molecules containing nitrogen, two nitrate transporters, Glyma.13G323800 (i.e., GmNRT2) [68] and Glyma.17G124900, were significantly down- and up-regulated in response to P deficiency, respectively, while one ammonium transporter was up-regulated in nodules (Figure 4), which suggests that P deficiency significantly influences N acquisition and translocation in nodules (Figure 4). Moreover, P deficiency led to significant increases in the transcription of three sugar transporters, two glycerol-3-phosphate transporters, two ascorbate transporters, one nucleoside transporter, one nucleobase-ascorbate transporter, and one organic cation/carnitine transporter, while decreases in the transcription of two aluminum-activated malate transporters in soybean nodules (Figure 4).

In regard to transport of other mineral nutrients, down-regulation was observed for two sulfate transporters and one boron transporter (Figure 4). On the other hand, up-regulation was observed for four sulfate transporters, two iron transporters, a single boron transporter, potassium transporter, and NRAMP metal ion transporter (Figure 4). These results suggest that P deficiency significantly influences the acquisition or translocation of many nutrients and metabolites in nodules.

### 2.7. Genes Involved in Hormonal Signaling Pathways

A total of 38 genes involved in hormonal signaling were differentially expressed in response to Pi starvation, which affected four types of hormone signaling pathways. These DEGs included 11 genes involved in auxin signaling, two in cytokinin (CK) signaling, 22 in ethylene signaling, and three in gibberellin (GA) signaling (Table 4). Furthermore, nine out of 11 auxin signaling DEGs, one out of two CK signaling DEGs, 19 out of 22 ethylene signaling DEGs, and all of the GA signaling DEGs were up-regulated by Pi starvation in nodules (Table 4). These results suggest that P deficiency affects growth and development in nodules partially through hormone signaling networks.

### 2.8. Ca^2+^ Signaling Related Genes in Soybean Nodules Regulated by P Deficiency

A total of 24 Ca^2+^ signaling related DEGs were identified in P-deficient soybean nodules, including two coding calmodulin-like proteins, three genes for annexins, two for calcium ATPases, four for calcium-dependent protein kinase, 12 for calcium-binding proteins, and one calmodulin-binding transcription activator (Table 5). Among these 24 Ca^2+^ signaling related DEGs, 19 were up-regulated and five were down-regulated, including one annexin gene, three genes coding for calcium-binding proteins, and one gene encoding a calcium-dependent protein kinase (Table 5).

### 2.9. Transcription Factors in Soybean Nodules Regulated by P Deficiency

**Figure 5:**
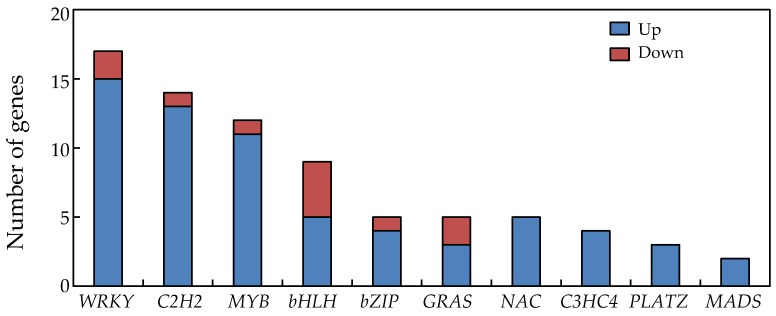
Differentially expressed genes associated with transcription factor activity in soybean nodules.

A total of 71 putative transcription factor genes responded significantly to P deficiency in soybean nodules (Figure 5). Among them, genes related to WRKY transcription factors were the most abundant, with 15 being up-regulated and two down-regulated (Figure 5). The remaining transcription factor DEGS were observed as follows. Thirteen *C2H2* family DEGs were up-regulated, but one was down-regulated, 11 *MYB* family DEGs were up-regulated and one down-regulated, five *bHLH* family DEGs were up-regulated and four down-regulated, four *bZIP* family DEGs were up-regulated and one down-regulated, and three *GRAS* family DEGs were up-regulated and two down-regulated (Figure 5). Interestingly, five *NAC* members, four *C3HC4* members, three *PLATZ* members, and two *MADS* members identified in nodules were up regulated by P deficiency (Figure 5).

### 2.10. Analysis of Gene Transcripts Using qRT-PCR

**Figure 6:**
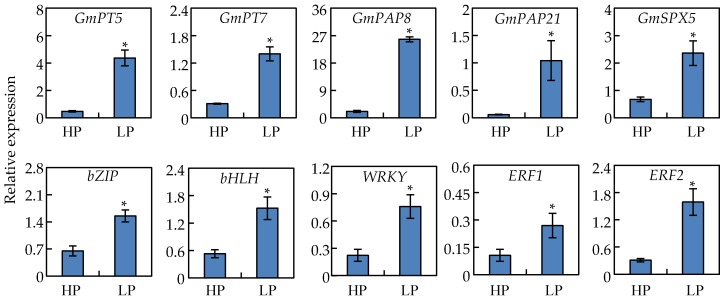
qRT-PCR analysis of ten Pi responsive genes in soybean nodules under Pi sufficient (HP) and deficient (LP) conditions. Data in the figure are mean of four replicates with standard error. Asterisks indicate significant difference between HP and LP treatments in the Student’s *t*-test (*: *p* < 0.05).

To confirm results from the RNA-seq analysis, qRT-PCR analysis was further conducted with 10 up-regulated DEGs in nodules at two P levels. All of these genes were significantly up-regulated by Pi starvation in soybean nodules as determined by qRT-PCR procedures, which strongly supports the reliability of the RNA-seq results (Figure 6 and Figure S3). Among the transcripts subjected to qRT-PCR analysis, respective increases of over 7-fold and 3-fold were observed for two Pi transporters genes, *GmPT5* and *GmPT7* (Figure 6). One SPX gene (*GmSPX5*), two purple acid phosphatase genes (*GmPAP8* and *GmPAP21*) exhibited 4-, 11-, and 17-fold increases, respectively, in LP plants relative to those in the HP treatment (Figure 6). In qRT-PCR analysis of the tested transcription factor DEGs, *bZIP* (Glyma.04G022100) was up-regulated more than 2-fold, *bHLH* (Glyma.11G043700) was up-regulated more than 2.8-fold, and the tested *WRKY* (Glyma.16G026400) was up-regulated about 3.3-fold in nodules subjected to P deficiency (Figure 6). Finally, the hormonal signaling ethylene response factors, *ERF1* (Glyma.01G206600) and *ERF2* (Glyma.04G022100) were up regulated by 2.5- and 2.3-fold, respectively (Figure 6).

## 3. Discussion

Leguminous plants form nodules through symbiotic interactions with rhizobium species. These organs are the sites of SNF, which provide nitrogen for host plants. However, it is well documented that P deficiency significantly influences nodule growth and development in legume plants, such as in soybean, common bean, *Medicago truncatula*, and chickpea [61,62,64,65,66]. Consistently, in this study, P deficiency also led to significant inhibition of nodule growth and development, as reflected by decreases in nitrogenase activity, nodule fresh weight and nodule size with Pi starvation (Figure 1). However, relative to effects on leaves and roots, decreases of total P content, soluble Pi concentration, and total P concentration in nodules were less affected by Pi starvation (Figure 1), strongly suggesting that nodules are P sinks with a high capability of maintaining Pi homeostasis to reduce adverse effects of P deficiency on nodule growth and development [60,62,65,66].

With the aid of genome-wide analysis of gene expression through microarray or RNA-seq approaches, a group of Pi starvation responsive genes have been identified in plant leaves and roots, which has facilitated the elucidation of adaptive strategies employed by plants to minimize the detrimental effects of P deficiency through functional characterization of these DEGs [11,69,70,71,72,73]. Recently, with the aid of RNA-seq approaches, genome wide analysis of Pi starvation responsive genes in legume nodules has been studied, with the host plants being common bean, *Medicago truncatula* and *chickpea* [64,65,66]. Common responses of legume nodules to Pi starvation could be demonstrated by identifying a set of DEGs with high homology among three legume species, such as *WRKY*, *MYB* and *NAC* [64,65,66]. However, it seems that more complex responses of soybean nodules to Pi starvation were elucidated as reflected by identification of 2055 Pi-starvation responsive genes, which was more than 495 in bean, 1140 in *Medicago truncatula* and 540 in chickpea [64,65,66]. For example, 8 *GmPT* members and *GmSPX* members were found to be responsive to Pi starvation in soybean nodules, but only 1 *SPX* member in *Medicago truncatula*, 1 *PT* member and *SPX* member in chickpea have been identified [64,65,66]. Furthermore, it seems that a set of genes preferring to increase transcripts in soybean nodules at low P levels were identified in the current study, such as *GmPT5*, *GmSPX1*, and *GmPAP11/30*. For example, among eight Pi-starvation up-regulated *GmSPX* members in soybean nodules, *GmSPX1* has been documented to exhibit no response to Pi starvation in soybean roots [74]. Meanwhile, transcription of *GmPAP11/30* was found to have no response to Pi starvation in soybean roots [27], suggesting complex responses of soybean nodules to Pi starvation.

Enhanced Pi mobilization and acquisition through increased exudation of organic acids and purple acid phosphatase, along with up-regulation of Pi transporters are well-documented strategies employed by plant roots in response to P deficiency [75,76,77,78,79,80,81]. Nodules exhibit similar responses to roots in response to P deprivation, with up-regulation of genes related to Pi mobilization and acquisition, such as Pi transporters, and purple acid phosphatases, which allows for the maintenance of Pi homeostasis in nodules (Table 3 and Figure 4). In this study, eight out of 14 *GmPT* members were significantly enhanced in nodules as a result of P deficiency (Table 3). Among them, Pi starvation up-regulated *GmPT5* might mediate Pi homeostasis in soybean nodules through control of Pi translocation from roots to nodules [35]. In the present study, other three *GmPT* members (i.e., *GmPT2*/*6*/*14*) were found to be up-regulated by Pi starvation, strongly suggesting other *GmPT* members could mediate Pi acquisition and translocation in soybean nodules at low P level except to *GmPT5* [62], which merits further analysis.

Accompanying increases in the abundance of nine *GmPT* transcripts, 16 *PAP* transcripts were also observed as differentially expressed in nodules subjected to Pi starvation, which is consistent with observations of significantly increased APase activity in P deprived nodules (Table 3 and Figure 2). Increased *PAP* transcription and APase activity are well known to play vital roles in the regulation of internal P metabolism and extracellular organic P mobilization in plants [82,83]. Although functions of several *GmPAP* have been documented, including the involvement of *GmPhy* and *GmPAP4* in phytate-P mobilization, and the participation of *GmPAP3* in ROS metabolism in plants under salt stress, functions of most Pi starvation up-regulated *GmPAPs*, except *GmPAP21*, remain largely unknown [84,85,86,87]. *GmPAP21* overexpression leads to nodule growth inhibition in soybean, suggesting that it participates in internal P metabolism within soybean nodules [87]. Furthermore, it was observed that organic-P utilization was enhanced in rhizobia inoculated in soybean, it is reasonable to hypothesize that Pi starvation responsive *GmPAPs* might also be involved in extracellular organic-P utilization in soybean [18]. Among Pi starvation up-regulated *GmPAPs*, GmPAP11/20/23 exhibited high homology with SgPAP10 in stylo functions as mediating extracellular organic-P utilization [81], suggesting that *GmPAP11*/20/23 might contribute to extracellular organic-P utilization in soybean nodules.

In addition to *GmPT* and *GmPAP*, two *GmSPX* genes, *GmSPX1* and *GmSPX3,* are also potentially vital regulators of Pi signaling pathways in soybean [74,88]. Interestingly, *GmSPX1* and *GmSPX3*, together with six other *GmSPX* members were found to be significantly up-regulated in soybean nodules upon Pi starvation (Table 3). This indicates that *GmSPX* members are good candidates for genes involved in maintaining Pi homeostasis in soybean nodules.

In addition to differential expression associated with Pi acquisition and mobilization, many Pi-starvation responsive DEGs in soybean nodules were associated with nitrate/nitrite absorption and assimilation (Figure S2). Similarly, Pi starvation can lead to significant increases in the concentrations of total amino acids and asparagine in common bean and chickpea [64,89]. Furthermore, consistent with increased asparagine accumulation, three *asparagine synthetase* genes were found to be up-regulated by Pi starvation in soybean nodules (Table 1 and Table S3), strongly suggesting that Pi starvation significantly influences amino acid accumulations in nodules. Increased asparagine accumulation is known to inhibit the capacity for SNF in nodules, suggesting that asparagine plays a role in N feedback regulation of SNF [90,91,92]. Plus, nitrogenase activity has been severely curtailed through phloem-feeding of asparagine, which further implicates asparagine as a phloem-mobile shoot-born factor that functions in systemic feedback regulation of SNF [91]. However, these previous investigations did not include experiments of P effects. Therefore, further investigation of regulatory mechanisms underlying amino acid synthesis and transport involving nodules in responses to Pi starvation remains as a relevant subject for future researchers.

In this study, 38 plant hormone-related genes were identified as DEGs in response to P deprivation (Table 4). This indicates that a variety of signaling pathways within nodules participate in responses to Pi starvation. For example, two genes (Glyma.10G056200 and Glyma.16G020800) coding auxin-responsive proteins were down-regulated, while one auxin responsive factor (Glyma.19G206100) and 5 AUX/IAA family members were up-regulated by Pi starvation in soybean nodules (Table 4). This suggests that auxin is involved in nodule adaptation to Pi starvation. However, specific roles for auxin signaling in adaptive strategies of nodules to Pi starvation remain unknown. Although miR160 can negatively regulate *AUXIN RESPONSE FACTOR10* (*ARF10*), and, thus, increase auxin sensitivity and inhibit soybean nodule development [93], none of these genes were found to be significantly regulated by Pi starvation in the present study (Table 4). Therefore, other auxin pathways might also regulate nodule responses to Pi starvation, which requires further investigation for more conclusive evidence.

Calcium signaling was also found to be important in the current work, as 24 Ca^2+^ signaling related genes were found to be regulated by Pi starvation in soybean nodules, including two *calmodulin-like* and four *calcium-dependent protein kinase*. This suggests that low P availability affects Ca^2+^ signaling, and thereby regulates nodule development (Table 5). Consistent with this result, sustained oscillation of calcium concentrations is known to activate the expression of symbiosis-related genes after perception of rhizobia-derived nodulation factors [94,95]. Meanwhile, CCaMK, a nuclear calcium- and calmodulin-dependent kinase has been suggested as the central regulator in symbiotic development in plants [96]. All these results strongly suggest that Ca^2+^ signaling is also involved in regulating soybean nodule adaptations to Pi starvation.

Finally, significant alterations of transcriptional regulation are implied by the presence of 76 transcription factors among the DEGs responsive to P deficiency in soybean nodules. These numbers include 12 MYB and five GRAS transcription factors (Figure 5). Although functions of MYB and GRAS transcription factors remains largely unknown in soybean nodule development and responses to Pi starvation, one MYB transcription factor, *LjIPN2*, has been documented as capable of binding directly to the *NIN* gene promoter and, thus, play an important role in the Nod signaling pathway in *Lotus japonicas* [97]. Meanwhile, it has been reported that the GRAS family transcription factors, *MtNSP1* and *MtNSP2* form a protein complex that is essential for root nodule symbiosis in *Medicago truncatula* [98]. The results herein are consistent with these previous reports and further suggest that complex transcriptional regulatory networks participate in soybean nodule adaption to Pi starvation.

## 4. Materials and Methods

### 4.1. Plant, Rhizobium and Growth Conditions

The soybean (*Glycine max* L.) genotype YC03-3 and rhizobium strain USDA110 were selected for hydroponic experiments. Seeds were sterilized and germinated in paper rolls for 4 days. Before transplanting, roots of uniform seedlings were inoculated with rhizobia. The nutrient solution contained KNO_3_ 311.3 μM, NH_4_NO_3_ 94.3 μM, MgCl_2_ 25 μM, MgSO_4_·7H_2_O 500 μM, K_2_SO_4_ 300 μM, MnSO_4_·H_2_O 1.5 μM, ZnSO_4_·7H_2_O 1.5 μM, CuSO_4_·5H_2_O 0.5 μM, (NH_4_)_5_MoO_24_·4H_2_O 0.16 μM, Fe-EDTA (Na) 40 μM, NaB_4_O_7_·10H_2_O 2.5 μM, and 25 μM KH_2_PO_4_ (LP) or 500 μM KH_2_PO_4_ (HP). The pH value was adjusted to approximately 5.8, and the nutrient solution was changed weekly. Leaves, roots, and nodules were harvested 25 days after transplanting.

### 4.2. Determination of Total P and Soluble Pi Concentrations

Total Pi and soluble Pi concentrations were analyzed as described previously [62]. For the total P concentration measurements, about 0.2 g dry weight of shoots, roots, and nodules from each P treatment was separately digested for each tissue in H_2_SO_4_, and further boiled and digested at 300 °C until the solution became clear. For soluble Pi concentrations, about 0.1 g of fresh samples of soybean leaves, roots, and nodules were sampled separately and ground in deionized water for extraction. The supernatant was collected after centrifugation at 12,000× *g* for 30 min. Total and soluble Pi concentrations were determined as described by Murphy and Riley [99].

### 4.3. Acid Phosphatase Activity Measurements

Acid phosphatase activities of leaves, roots, and nodules were assessed as described previously [77]. Briefly, about 0.1 g of fresh samples were ground and extracted for soluble protein using 100 mM Tris-HCl (pH 8.0). Reaction mixtures containing 1 mM ρ-nitrophenyl phosphate (ρ-NPP, Sigma, Saint Louis, MO, USA), 2 mL of 45 mM Na-acetate buffer (pH 5.0) and protein extract were incubated at 37 °C for 15 min before halting reactions via the addition of 1 mL of 1 M NaOH. Absorbance was measured at 405 nm. The concentration of soluble protein was analyzed using Coomassie Brilliant Blue staining [100]. Acid phosphatase activity was presented as micromoles of ρ-NPP hydrolyzed per milligram of protein per minute.

### 4.4. Nodule Nitrogenase Activity Analysis

Nodules attached roots were cut off to measure nitrogenase activity by the acetylene reduction assay [101]. Fresh nodules were incubated in a closed container with 10% (*v*/*v*) acetylene gas for 2 h at 28 °C prior to extracting 1 mL of reacted gas samples from the headspace using a syringe. Ethylene content was calculated from peak areas of standards analyzed by gas chromatography. Nitrogenase activity was calculated as μmole ethylene h^−1^·g^−1^ nodules.

### 4.5. Amino Acid Analysis

The amino acid compositions of nodules were analyzed in an automatic amino acid analyzer (type L-8800, Hitachi Ltd., Tokyo, Japan), as described previously [102]. Briefly, about 0.1 g of fresh samples were cut off and ground in 5% 5-sulfosalicylic acid dehydrated for the extraction. The supernatant was collected after centrifugation at 12,000× *g* at 4 °C for 30 min. Separation column (4.6 mm × 60 mm) parameters: eluent flow rate = 0.4 mL/min, column temperature = 70 °C, and column pressure = 11.627 MPa. Reaction column parameters: ninhydrin buffer run at a flow rate of 0.35 mL/min, column temperature = 135 °C, column pressure = 1.078 MPa. The detection threshold for amino-N compounds was 1 µg·g^−1^ fresh weight in nodules.

### 4.6. cDNA Library Preparation, RNA-Seq and Phylogenetic Analysis

After 25 days, nodules with the size more than 3 mm were collected for mRNA library construction and sequencing. Nodules from two plants were pooled together as one replicate, and three replicates from each P treatment were used for RNA-seq analysis. Total RNA of nodules was isolated using Trizol reagent (Invitrogen, Carlsbad, CA, USA) according to the manufacturer’s protocol. The quantity and purity of total RNA was checked using an Agilent 2100 RNA Nano 6000 Assay Kit (Agilent Technologies, Palo Alto, CA, USA). About 3 μg of total RNA was subjected to mRNA enrichment using oligo (dT) attached magnetic beads (Invitrogen). The mRNA was then fragmented into small pieces using fragmentation buffer, which was then used as template strands. The first strand of cDNA was synthesized by random hexamers, then added buffer, dNTPs, RNase H, and DNA polymerase I was added for synthesizing the second chain. Through QiaQuick PCR amplification, purification and EB buffer elution, end repair, addition of adenosine and sequencing joints, target size fragments were recovered by agarose gel electrophoresis prior to performing and PCR amplification. Sequencing was performed on the Illumina HiSeq X Ten platform, and the sequencing program was PE150. To obtain clean reads, the adaptor sequences, low quality sequences, and unknown nucleotides were removed from the raw reads. Bowtie2 software (version 2.2.3) was used for building the genome index, and clean reads were then aligned to the reference genome (*Glycine max* Williams82.a2.v1) using HISAT2 software (version 2.1.0) with no more than one nucleotide mismatch allowed, the alignment results were identified and estimated through Cufflinks (version 2.2.0) [103]. Gene expression levels were normalized through fragments per kilobase million mapped reads (FPKM) method [104] and are shown in Table S5. DESeq2 v1.6.3 was designed for differential gene expression analysis between P-deficient and P-sufficient nodules. The expression level of each gene per sample was estimated by the linear regression, then the *p*-value was calculated with Wald test, finally the *p*-value was corrected by the BH method, and genes with *q* ≤ 0.05 and |log_2_(LP/HP)| ≥ 1 were identified as differentially expressed genes (DEGs) [105]. GO functional enrichment analysis using the DAVID (Database for Annotation, Visualization and Integrated Discovery) [106]. Visualization of metabolic pathways was achieved using MapMan software [107]. Phylogeny analysis was conducted by MEGA 5.05, using the neighbor-joining method with 1000 bootstrap replicates as described previously [108]. All the original RNA-Seq data have been submitted to the NCBI Gene Expression Omnibus under the accession number of GSE116593.

### 4.7. qRT-PCR Analysis

Total RNA from nodules was separately extracted using Trizol reagent (Invitrogen, Carlsbad, CA, USA). After treating with DNase I, the reverse transcription kit (Promega, Madison, WI, USA) was used to synthesize first-strand complementary DNA. The qRT-PCR analysis was performed by SYBR Green monitored qPCR (Takara, Kyoto, Japan) and carried out on a Rotor-Gene 3000 qPCR system (Corbett Research, Mortlake, Australia), with the following reaction conditions: 95 °C for 30 s, 40 cycles of 95 °C for 5 s, 60 °C for 15 s, and 72 °C for 30 s. Three biological replications were included. Relative expression levels were calculated as the ratio of candidate gene expression to housekeeping gene *TefS1* (Glyma.17G186600) expression as described previously [51]. The primers for qRT-PCR analysis are shown in Table S6.

### 4.8. Statistical Analysis

Statistical analysis was performed using Microsoft Excel 2010 (Microsoft Company, Redmond, WA, USA). Significant differences between treatments were evaluated by statistical comparison performed using Student’s *t*-test.

## 5. Conclusions

Phosphorus deficiency severely inhibited soybean nodule growth and symbiotic nitrogen fixation. However, soybean nodules exhibited a superior capability of maintaining Pi homeostasis, as reflected by smaller effects of Pi starvation on nodule Pi concentration than in either soybean leaves or roots. With the aid of genome wide RNA-seq analysis, a total of 2055 genes were identified as differentially expressed genes between Pi sufficient and deficient conditions, suggesting that multiple complex transcriptional regulatory networks act in soybean nodule adaption to Pi starvation. Furthermore, a set of DEGs can be associated though annotations and previous work with maintenance of nodule Pi homeostasis. This set includes eight *PTs*, 8 *SPXs*, and 16 *PAPs*, which further supports the conclusion that multiple regulatory pathways are involved in maintaining Pi homeostasis through effects on Pi acquisition, translocation, and mobilization in soybean nodules. Taken together, this dataset will be valuable for further efforts to elucidate molecular mechanisms underlying soybean nodule adaption to P deficiency.

## Figures and Tables

**Table 1:** Amino acid composition in soybean nodules under phosphate (Pi) sufficient (HP, 500 μM KH_2_PO_4_) and deficient (LP, 25 μM KH_2_PO_4_) conditions.

	HP		LP		Log_2_(LP/HP)
	µg/g FW	%	µg/g FW	%	
Asparagine	3225.00 ± 570.99	69.47	5039.99 ± 720.71	79.83	0.64 *
Glutamic acid	619.90 ± 45.82	13.35	285.56 ± 32.07	4.52	−1.12 *
γ-Aminobutyric acid	148.83 ± 9.96	3.21	134.97 ± 23.90	2.14	−0.14
Serine	118.56 ± 9.95	2.55	117.30 ± 0.88	1.86	−0.02
Aspartic acid	107.75 ± 8.35	2.32	68.12 ± 8.58	1.08	−0.66 *
Alanine	62.91 ± 4.19	1.36	53.39 ± 22.40	0.85	−0.24
Tryptophane	51.22 ± 3.52	1.10	42.19 ± 5.36	0.67	−0.28
Glutamine	43.98 ± 53.87	0.95	84.27 ± 33.24	1.33	0.94
Arginine	43.30 ± 4.81	0.93	155.11 ± 12.52	2.46	1.84 *
β-Alanine	39.17 ± 4.00	0.84	30.68 ± 2.15	0.49	−0.35 *
Phosphoserine	34.65 ± 1.88	0.75	33.11 ± 2.49	0.52	−0.07
Histidine	30.23 ± 3.20	0.65	135.99 ± 7.34	2.15	2.17 *
Threonine	23.54 ± 3.23	0.51	22.33 ± 2.22	0.35	−0.08
Phenylalanine	18.79 ± 2.42	0.40	17.25 ± 1.68	0.27	−0.12
Tyrosine	12.91 ± 4.17	0.28	11.63 ± 1.44	0.18	−0.15
Lysine	12.10 ± 1.71	0.26	13.32 ± 1.72	0.21	0.14
Citrulline	10.17 ± 4.09	0.22	7.28 ± 1.69	0.12	−0.48
Glycine	8.31 ± 1.29	0.18	8.86 ± 0.66	0.14	0.09
Isoleucine	5.92 ± 0.55	0.13	10.24 ± 0.38	0.16	0.79 *
Cystine	5.89 ± 0.75	0.13	3.90 ± 1.28	0.06	−0.59 *
Leucine	4.89 ± 1.48	0.11	5.49 ± 0.94	0.09	0.17
Valine	4.71 ± 0.83	0.10	3.48 ± 0.33	0.06	−0.44
Ornithine	3.54 ± 1.20	0.08	2.52 ± 0.78	0.04	−0.49
Methionine	3.18 ± 0.09	0.07	2.25 ± 1.18	0.04	−0.5
Phosphorylethanolamine	2.64 ± 1.13	0.06	24.30 ± 0.56	0.38	3.20 *
Total	4642.09	100	6313.53	100	

The fold change of the amino acid concentration between HP and LP conditions was calculated as the logarithm of LP/HP to the base 2 (i.e., log_2_(LP/HP)). Data in the table are means of three replicates with standard error. Asterisks indicate significant difference between HP and LP treatment (*: *p* < 0.05).

**Table 2:** Gene number identified through RNA-seq analysis.

	Total Expressed Genes	Up-Regulated	Down-Regulated
HP	38,813		
LP	38,874		
DEGs *	2055	1431	624

* DEGs: Differentially expressed genes between Pi sufficient (HP) and deficient (LP) soybean nodules.

**Table 3:** Differentially expressed genes involved in Pi homeostasis.

Accession No.	Name/Description	Log_2_(LP/HP)	*Q* Value
Glyma.02G005800	*GmPT1*	1.15	3.0 × 10^−2^
Glyma.03G162800	*GmPT2*	1.41	1.34 × 10^−15^
Glyma.10G006700	*GmPT4*	3.99	1.12 × 10^−45^
Glyma.10G036800	*GmPT5*	4.04	2.84 × 10^−166^
Glyma.10G186400	*GmPT6*	4.01	2.56 × 10^−30^
Glyma.10G186500	*GmPT7*	2.28	5.85 × 10^−50^
Glyma.20G204000	*GmPT13*	1.82	1.3 × 10^−13^
Glyma.20G204100	*GmPT14*	3.67	1.02 × 10^−21^
Glyma.02G117000	*GmPAP1*	2.15	3.94 × 10^−15^
Glyma.05G138400	*GmPAP8*	3.03	3.05 × 10^−43^
Glyma.05G247900	*GmPAP9*	2.45	5.49 × 10^−93^
Glyma.05G247800	*GmPAP10*	2.28	4.15 × 10^−29^
Glyma.06G028200	*GmPAP11*	5.41	1.90 × 10^−239^
Glyma.08G056400	*GmPAP13*	2.43	1.11 × 10^−84^
Glyma.08G093500	*GmPAP15*	2.19	2.92 × 10^−46^
Glyma.08G093600	*GmPAP16*	2.29	8.75 × 10^−47^
Glyma.08G291600	*GmPAP17*	1.70	2.33 × 10^−19^
Glyma.09G229200	*GmPAP20*	3.70	3.36 × 10^−162^
Glyma.10G071000	*GmPAP21*	4.61	9.92 × 10^−78^
Glyma.12G007500	*GmPAP23*	3.14	3.07 × 10^−110^
Glyma.18G132500	*GmPAP30*	1.29	3.01 × 10^−13^
Glyma.19G026600	*GmPAP31*	4.86	1.32 × 10^−73^
Glyma.19G193900	*GmPAP32*	1.81	2.24 × 10^−4^
Glyma.20G026800	*GmPAP35*	1.66	1.02 × 10^−35^
Glyma.01G135500	*GmSPX1*	6.35	0
Glyma.04G067400	*GmSPX2*	2.09	1.39 × 10^−5^
Glyma.04G147600	*GmSPX3*	4.55	9.01 × 10^−105^
Glyma.06G069000	*GmSPX4*	5.89	5.27 × 10^−124^
Glyma.10G261900	*GmSPX5*	1.99	2.31 × 10^−14^
Glyma.13G166800	*GmSPX7*	3.41	4.79 × 10^−103^
Glyma.17G114700	*GmSPX8*	3.52	1.03 × 10^−73^
Glyma.20G129000	*GmSPX9*	7.40	2.6 × 10^−118^

**Table 4:** Differentially expressed genes involved in hormonal signaling.

Hormone	Accession No.	Name/Description	Log_2_(LP/HP)	*Q* Value
Auxin	Glyma.02G142400	AUX/IAA family auxin-responsive protein	2.25	3.56 × 10^−8^
	Glyma.02G142500	AUX/IAA family auxin-responsive protein	2.28	5.80 × 10^−16^
	Glyma.04G025300	AUX/IAA family auxin-responsive protein	1.91	3.46 × 10^−24^
	Glyma.05G196300	AUX/IAA family auxin-responsive protein	1.58	4.80 × 10^−7^
	Glyma.09G193000	AUX/IAA family auxin-responsive protein	1.06	3.69 × 10^−2^
	Glyma.10G031900	SAUR family auxin-responsive protein	1.86	7.77 × 10^−6^
	Glyma.10G056200	SAUR family auxin-responsive protein	−1.07	9.92 × 10^−4^
	Glyma.12G035700	SAUR family auxin-responsive protein	1.84	5.57 × 10^−5^
	Glyma.13G361200	SAUR family auxin-responsive protein	1.06	2.09 × 10^−6^
	Glyma.16G020800	SAUR family auxin-responsive protein	−1.27	1.70 × 10^−2^
	Glyma.19G206100	Auxin response factor	2.16	7.42 × 10^−6^
Cytokinin	Glyma.04G055600	Cytokinin dehydrogenase	−1.25	2.96 × 10^−3^
	Glyma.20G159600	Cytokinin hydroxylase	1.56	1.95 × 10^−3^
Ethylene	Glyma.01G206600	AP2-like ethylene response factor	1.18	3 × 10^−2^
	Glyma.02G132500	AP2-like ethylene response factor	1.90	2.06 × 10^−5^
	Glyma.05G063500	AP2-like ethylene response factor	1.12	3.03 × 10^−4^
	Glyma.06G236400	AP2-like ethylene response factor	1.09	6.61 × 10^−6^
	Glyma.07G113800	AP2-like ethylene response factor	1.28	4.46 × 10^−3^
	Glyma.07G212400	AP2-like ethylene response factor	2.60	1.05 × 10^−18^
	Glyma.09G072000	AP2-like ethylene response factor	2.30	2.54 × 10^−10^
	Glyma.10G118900	AP2-like ethylene response factor	1.01	7.27 × 10^−6^
	Glyma.10G194200	AP2-like ethylene response factor	1.23	6.20 × 10^−3^
	Glyma.12G110400	AP2-like ethylene response factor	1.04	4 × 10^−2^
	Glyma.12G203100	AP2-like ethylene response factor	1.32	3.03 × 10^−4^
	Glyma.13G040400	AP2-like ethylene response factor	−1.13	2 × 10^−2^
	Glyma.13G112400	AP2-like ethylene response factor	2.16	1.95 × 10^−6^
	Glyma.15G180000	AP2-like ethylene response factor	2.87	3.81 × 10^−16^
	Glyma.17G047300	AP2-like ethylene response factor	1.20	3 × 10^−2^
	Glyma.17G070800	AP2-like ethylene response factor	1.25	6.47 × 10^−3^
	Glyma.17G145300	AP2-like ethylene response factor	1.26	1.54 × 10^−5^
	Glyma.18G281400	AP2-like ethylene response factor	−1.35	3.86 × 10^-4^
	Glyma.19G256800	AP2-like ethylene response factor	1.27	2 × 10^−2^
	Glyma.20G070100	AP2-like ethylene response factor	1.93	2.56 × 10^−9^
	Glyma.20G172800	AP2-like ethylene response factor	−1.18	2 × 10^−2^
	Glyma.14G041500	EIN3-like ethylene response factor	1.32	8.33 × 10^−19^
Gibberellin	Glyma.12G137700	Gibberellin-responsive protein	1.44	7.13 × 10^−4^
	Glyma.13G285400	Gibberellin-responsive protein	1.49	2.82 × 10^−8^
	Glyma.19G013000	Gibberellin-regulated protein	1.06	3 × 10^−2^

**Table 5:** Differentially expressed genes involved in Ca^2+^ signaling.

Accession No.	Name/Description	Log_2_(LP/HP)	*Q* Value
Glyma.05G085200	Annexin	1.86	8.71 × 10^−9^
Glyma.05G178200	Annexin	1.18	7.92 × 10^−16^
Glyma.10G002200	Annexin-like	−1.29	4.65 × 10^−14^
Glyma.01G166100	Calmodulin-binding transcription activator	1.76	2.47 × 10^−17^
Glyma.02G059200	Calcium-transporting ATPase	1.66	4.21 × 10^−13^
Glyma.02G245700	Calcium-transporting ATPase	1.44	4.46 × 10^−13^
Glyma.03G138000	Calmodulin-like protein	−1.03	3.38 × 10^−4^
Glyma.04G064800	Calmodulin-like protein	1.43	3.68 × 10^−5^
Glyma.04G136200	Calcium-binding protein	1.32	1.40 × 10^−4^
Glyma.05G047100	Calcium uptake protein	1.15	2 × 10^−2^
Glyma.05G199400	Calcium-binding protein	1.47	5.09 × 10^−4^
Glyma.06G171100	Calcium-binding protein	1.06	4 × 10^−2^
Glyma.07G229500	Calcium-binding protein	1.56	8.46 × 10^−6^
Glyma.08G006900	Calcium-binding protein	−1.19	5.63 × 10^−3^
Glyma.11G048300	Calcium-binding protein	1.50	2.93 × 10^−4^
Glyma.11G077300	Calcium-binding protein	1.16	3.34 × 10^-3^
Glyma.11G157100	Calcium-binding protein	1.11	2 × 10^−2^
Glyma.12G217700	Calcium-binding protein	2.23	1.37 × 10^−9^
Glyma.14G156300	Calcium-binding protein	−1.48	2.72 × 10^−3^
Glyma.14G222000	Calcium-binding protein	1.58	2.06 × 10^−20^
Glyma.16G142100	Calcium-binding protein	1.12	1.60 × 10^−9^
Glyma.17G128900	Calcium-dependent protein kinase	1.40	1.65 × 10^−6^
Glyma.20G034200	Calcium-dependent protein kinase	1.06	1.73 × 10^−4^
Glyma.20G066800	Calcium-dependent protein kinase	1.55	9.09 × 10^−16^
Glyma.05G248000	Calcium-dependent protein kinase	−1.08	2.08 × 10^−4^

## Supplementary Materials

Supplementary materials can be found at http://www.mdpi.com/1422-0067/19/10/2924/s1, References [109,110,111,112,113] were referred to in supplementary Figures S4 and S6.

## Abbreviations

SNF	Symbiotic Nitrogen Fixation
RNA-seq	RNA sequencing
DEGs	Differentially Expressed Genes
qRT-PCR	quantitative Real-Time Polymerase Chain Reaction
GO	Gene Ontology
CK	Cytokinin
GA	Gibberellin
CHO	Carbohydrate
