# Supplementary material for: Genome Wide Transcriptome Analysis Reveals Complex Regulatory Mechanisms Underlying Phosphate Homeostasis in Soybean Nodules
Source: Int J Mol Sci. 2018 Sep 26;19(10):2924. doi: 10.3390/ijms19102924 (PMC6213598; doi:10.3390/ijms19102924)
Supplement: Supplementary file 1 [file ijms-19-02924-s001.zip › Supplementary files/Supplementary figures.pdf]

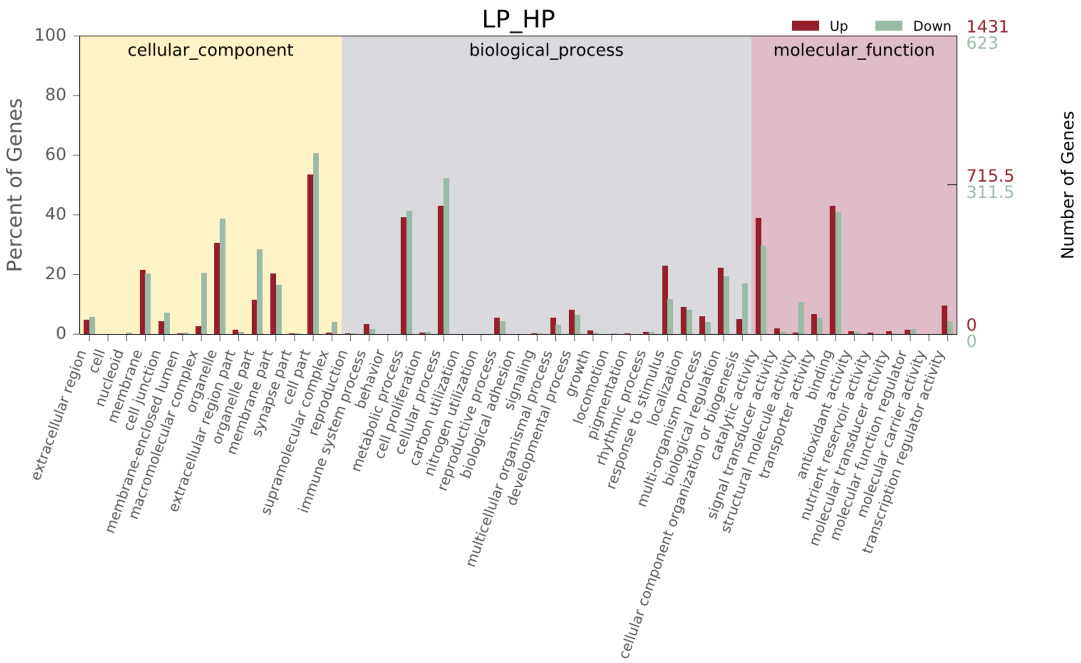

Figure S1. GO analysis of differentially expression genes.

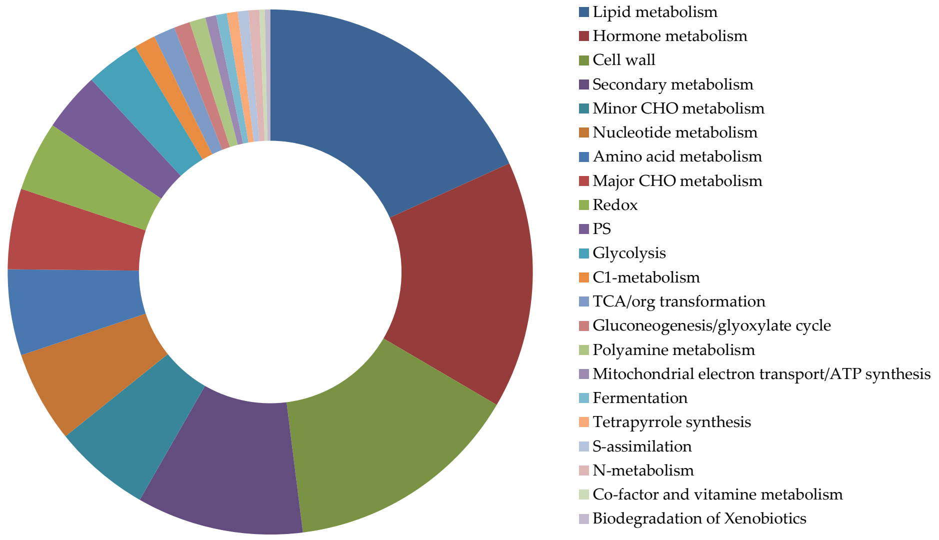

Figure S2. Differentially expressed genes involved in metabolic processes.

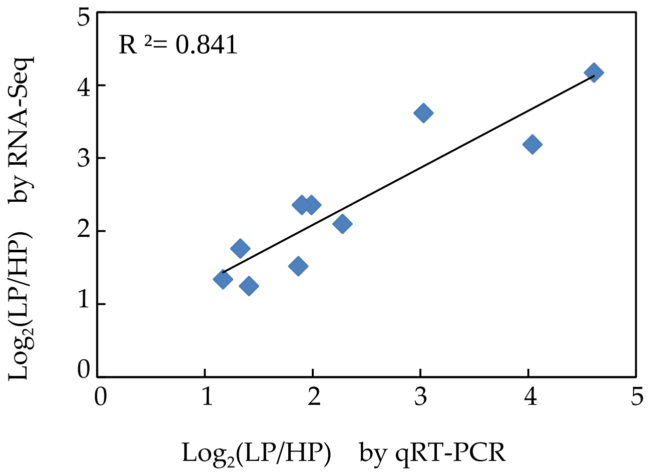

Figure S3. Correlation of expression between RNA-seq and qRT-PCR analyses for 10 selected genes up-regulated in response to P deprivation.

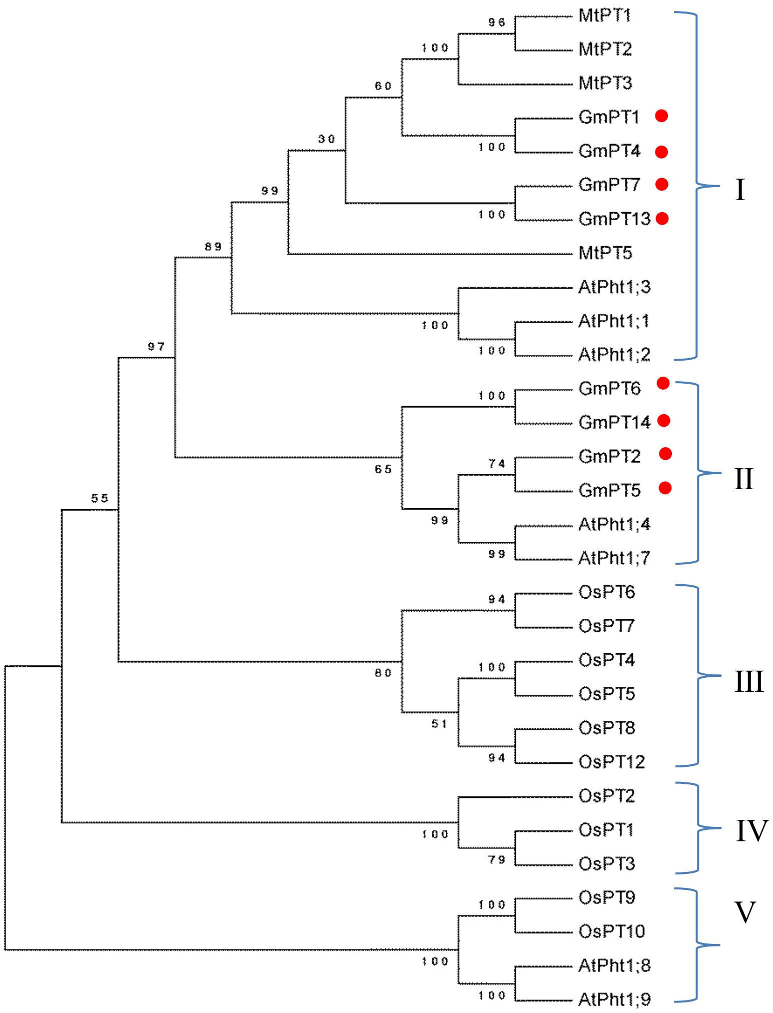

Figure S4. Phylogeny analysis of Pi transporter proteins. At, *Arabidopsis thaliana* [31]; Gm, *Glycine max* [28]; Os, *Oryza sativa* [109]; Mt, *Medicago truncatula* [110]. Red dots represent identified Pi-starvation responsive GmPTs in the present study. The phylogenetic tree was created using the Mega 5.05 program.

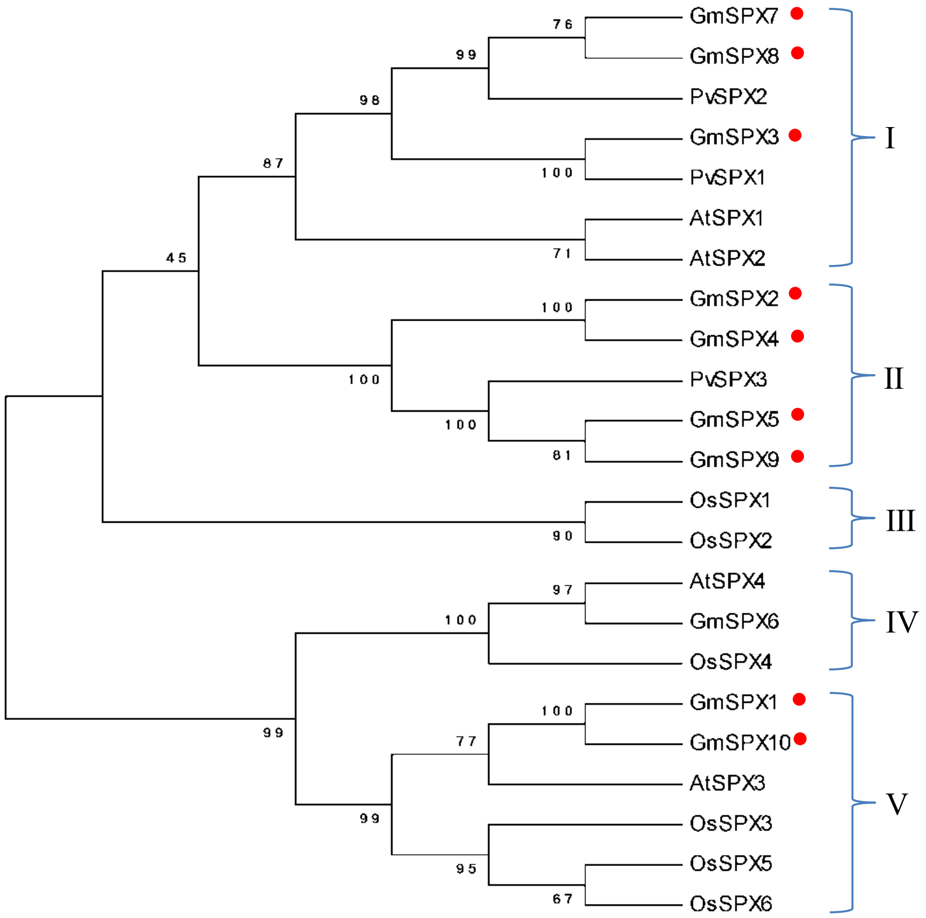

Figure S5. Phylogeny analysis of SPX proteins. At, *Arabidopsis thaliana* [22]; Gm, *Glycine max* [74]; Os, *Oryza sativa* [40]; Pv, *Phaseolus vulgaris* [24]. Red dots represent identified Pi-starvation responsive GmSPXs in the present study. The phylogenetic tree was created using the Mega 5.05 program.

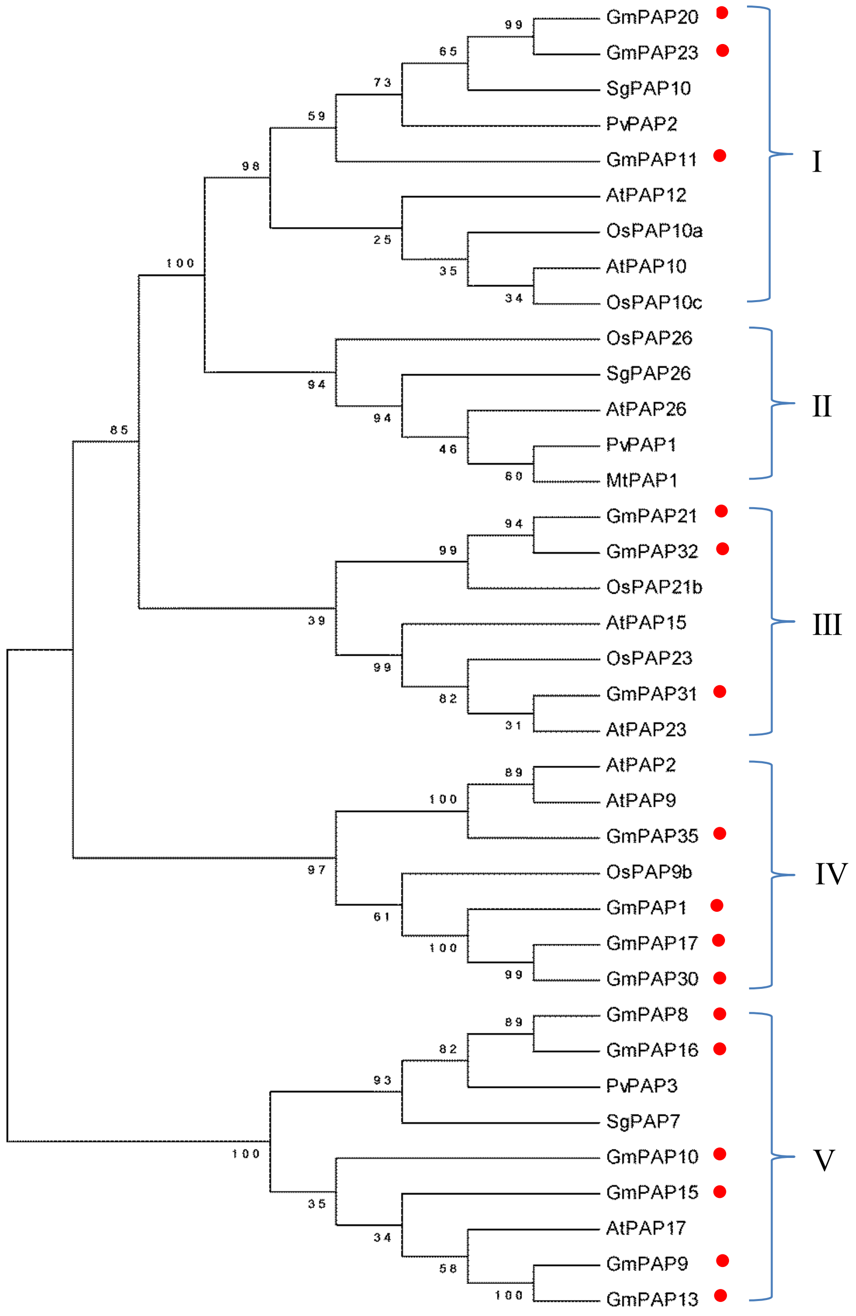

Figure S6. Phylogeny analysis of purple acid phosphatase (PAP) proteins. At, *Arabidopsis thaliana* [111]; Gm, *Glycine max* [27]; Os, *Oryza sativa* [112]; Pv, *Phaseolus vulgaris* [77]. Mt: *Medicago truncatula* [113]; Sg: *Stylosanthes guianensis* [81]. Red dots represent identified Pi-starvation responsive GmPAPs in the present study. The phylogenetic tree was created using the Mega 5.05 program.
